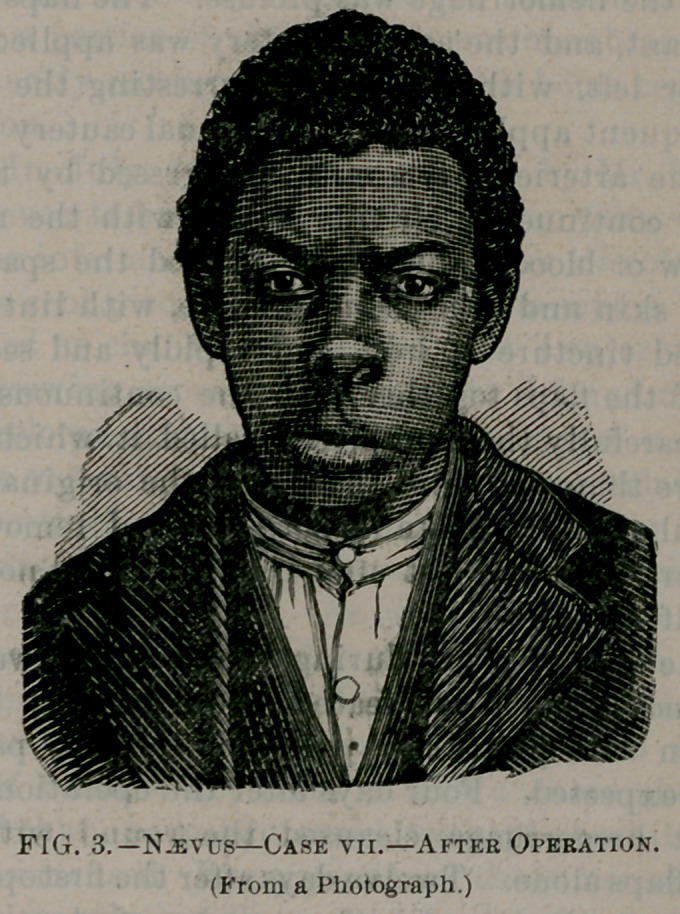# Nævus—With Reports of Cases

**Published:** 1884-09

**Authors:** W. F. Westmoreland

**Affiliations:** Atlanta, Ga.; Professor of the Principles and Practice of Surgery in the Atlanta Medical College


THE ATLANTA
MEDICAL AND SURGICAL JOURNAL.

VOL. I. SEPTEMBER, 1884. NO. 7.

### Original Communications.

### NÆVUS—WITH REPORT OF CASES.*

BY W. F. WESTMORELAND, M.D., ATLANTA, GA.

Professor of the Principles and Practice of Surgery in the Atlanta Medical College.

I do not propose in this paper to discuss the different varieties of nævi, their special pathology, mode of extension, etc., as this feature of the subject is well understood; but rather to impress the profession with the great importance of early and prompt attention to those forms of vascular growth requiring active surgical interference; and to illustrate its importance by the report of a few cases out of the many that have been submitted to me for treatment.

Practically, nævi may be divided into two distinct forms; in one the disease is stationary, rarely changing its shape, color or size without some accidental circumstance. To this class belong those wide-spread, diffuse or superficial cutaneous nævi—sometimes covering extensive continuous cutaneous surfaces – known as port-wine spots, spider marks, strawberry marks, etc., taking its name from the color or the peculiar shape of the discoloration. Usually no treatment is required, or I should rather say, no treatment yet proposed has been sufficiently successful in removing the wide-spread discoloration, to give confidence to the profession. The other form, the one so frequently requiring the attention of the surgeon, and which we feel should rather be called vascular tumors, or vascular growths, is by pathologists divided into three varieties—capillary,

*Read before the Medical Association of Georgia.

arterial and venous—the varieties dependent upon the predominance of the class of vessels forming the vascular growth. While these divisions are usually readily recognized, still, when the growth has existed for a length of time, or has been subjected to injuries followed by frequent hemorrhages and inflammations, or has reached great dimensions, it is often difficult to decide which set of vessels originally predominated. In the majority of cases, however, it makes but little difference which variety we have so far as treatment is concerned, if we are called before the tumor has reached any great dimensions; and often, even then, we adopt the same procedure, whether we have the capillary, arterial or venous varieties.

The great consideration, and the one I so much desire to impress, is the great importance of at once destroying the growth, by some of the modes to be suggested later, just as soon as it is determined that the nævus has commenced growing.

My experience is that the history of this form of vascular tumor is all about the same. At birth, or soon after, a mother's-mark, as they are often called, is detected, which for a longer or shorter time remains apparently stationary, sooner or later,—sometimes in a few weeks—again, years elapsing,— the little red or purple mark commences to increase more or less rapidly, and if left to itself, sooner or later reaching dimensions and producing deformities hideous in appearance, and often remaining a constant menace to the life of the subject of the trouble. It does appear cruel in the extreme to the little sufferer that after it has been made evident that the vascular spot is increasing in size—has commenced its growth—at a time when a slight operation, attended with no danger, would promptly relieve the trouble with slight or no deformity, to sit idly by and watch its continuous growth until it has reached dimensions which make an operation most difficult and dangerous, and after the best has been done and life saved, a needless and often a hideous deformity is the result of the delay. This, however, the surgeon cannot always control. In more than one case I have been forced, month after month, and in one case, year after year, to watch the more or less rapid growth, without being able, with all the eloquence and earnestness at my command, to impress the parents of the child with the importance of immediate surgical interference. One of the great difficulties in the way of inducing parents to submit their children to treatment, is the fact that for a time, or at least until the tumor has reached considerable dimensions, there is no pain attending the growth nor other interference with the general health of the subject. Again, it sometimes occurs that the growth is so slow that parents are not conscious of the fact that it is continuous. I have a case now under my observation, one, however, of very slow growth, in which the nævus is more than double the size it was twelve months ago, and still the mother is firmly of the opinion and candidly believes that the little tumor has not at all changed in size.

False or misdirected sympathy, after the judgment is convinced, sometimes leads to delays which result in permanent deformity, and entail great and unnecessary suffering upon the little patients. Twelve or fourteen years ago, I was consulted in two cases within a few months of each other. The children were about the same age, and both had a nævus, about the same size, upon the border of the upper lip. One was submitted at once to treatment, and with two needles at white heat the vessel was destroyed in a few seconds, and the child recovered with a slight cicatrix, which a few years later was hardly perceptible.

The mother of the other child would not permit her "little darling *to be punished*" in any such way, and left the city. One year later she returned to consult me, and I was really shocked at the rapidity of its growth and the deformity that the child presented. A little net-work of vessels which one year before was not much larger than half of a pea, had grown to the dimensions of a walnut with a subcutaneous extension under the right ala of the nose in the direction of the eye, and had reached a point midway between ala and the eye. Even in this condition she would not have submitted the child to treatment but for an alarming hemorrhage, the result of a fall which ruptured some of the vessels of the tumor. The child was relieved after three operations, each one of which produced fifty times the amount of suffering that the operation one year before would have produced, and worse still, the mistake of the mother in refusing an early operation entailed upon the child a permanent deformity, with a partial loss of the power to close the lower eye-lid, which will entail more or less annoyance as long as the child lives. I have but one rule when consulted in such cases. I first attempt to determine whether the nævus is stationary or increasing in size. If growing, I unhesitatingly insist upon active treatment for the arrest of development. If the growth is rapid it is readily determined by its feel and appearance. If, however, the growth is slow or in its incipiency, it is more difficult, and we are forced to rely upon the history of the case, the statements of the attendants, or better still, to wait a few days, or weeks that we may judge for ourselves.

In many cases where we are in doubt as to the growth of the nævus, particularly if the patient is so situated that it cannot conveniently be seen every few weeks, and the spot is small, and in a locality where deformity would result from a slight growth, I feel that we are not only justifiable in active interference, but regard it as the better course. I have, in several cases, operated under such circumstances, and in no instance have I had cause to regret it.

The different plans that have been proposed for the cure of this trouble are so numerous that I will barely have space to do more than allude to the majority of them ; each plan, however, having one of two objects, either the complete disorganization of the net-work of vessels constituting the tumor, or their complete extirpation, either with the bistoury, ligature or cautery.

When the nævus is small and confined to the true skin, that is before the commencement of the subcutaneous development, it may be destroyed in various ways, and often by the most simple means. Vaccination, when the virus is inserted into the discolored spots, is frequently sufficient to destroy the little net-work of vessels. One of the difficulties, however, in using this as a means of relief, is the great tendency of nævi to bleed when incised. The little incision made to introduce the virus often bleeds sufficiently freely to wash out the virus, and thus both vaccination and treatment have failed.

In such cases I have found much more reliable, from one to a half dozen punctures, the number dependent upon the sizeof the nævus, with needles at white heat. This is almost universally successful in destroying the vessels, and without the suffering that many would suppose to be the result of such treatment. Others use the various caustics, as nitric acid, ammonia, the preparations of zinc, etc., etc., but my experience is they are by no means so reliable, and then, the suffering to the patient is much greater than from the actual cautery.

Small threads of silk, flax or cotton immersed in the preparations of iron, as the perchloride or commercial tincture, or other astringent substances, and passed through these small nævi, and allowed to remain until inflammatory action is set up, often consolidate the vessels and arrest the development. Various other plans of treatment have, from time to time, been proposed, but one or the other of those above suggested will, I am sure, be found sufficient to effect the desired object.

After the growth becomes well marked, and the tumor has extended itself to the subcutaneous tissue, or in cases where they have their origin beneath the skin, or in parts still more deeply seated, there are many things to be taken into consideration before deciding upon the best plan to be adopted in each individual case.

The plans of treatment most frequently suggested are: the complete removal of the entire tumor by excision or by the ligature; the destruction of the mass, either by the actual or potential cautery; the use of injections into the vascular growth, as the preparations of iron, for the purpose of arresting the development of, and consolidating the vessels. The locality, size and shape of the vascular tumor, its relation to other tissue, the extent that the true skin is involved, and the class of vessels that predominate in the growth, are all to be well considered before we can properly decide as to the best plan to be adopted in any particular case.

Excision of the entire mass when practicable has many advocates as the safest, most rapid, least painful and the very best of all the plans proposed. There are, however, so many conditions that make excision impracticable, that in more than half the cases it cannot be considered If the nævus is irregular, with extensions and projections so frequently seen, or if it is in a locality where the complete loss of tissue would result in great deformity, or where we have the arterial variety (true aneurism by anastomosis), cases in which numerous tortuous and enlarged arteries constitute the greater portion of the tumor, we cannot in such cases think of excision. In fact, excision should only be attempted under peculiar circumstances and in connection with some other mode, unless our incision can be made in the normal tissue, outside of the diseased vessels. If we can do this, everything else being equal, it is certainly the very best plan, there being no more danger from hemorrhage than from an incision in like tissue in any other locality. The ligature has many advocates, and when practicable, fills the indications most perfectly; but, like excision, there are many cases, perhaps the majority, in which it is not applicable. The actual cautery is applicable in a greater number of cases than any other mode; and although there has been, from time to time, a great prejudice against its use, particularly in certain localities, still it is oftener used to-day than any one plan ever suggested. In connection with excision, it is particularly applicable in those formidable vascular tumors that I will allude to in the report of cases.

The potential cautery should, in my opinion, only be used in the very small nævi. In those large vascular growths they are very unreliable, and if great care be not exercised, serious or even fatal accidents may result.

I have never tried the injection of the perchloride of iron except in small, vascular tumors, and in the majority of the cases have succeeded in consolidating the vascular mass. Compression should only be attempted in cases where the tumor appears over the hard substance, as the integuments of the cranium. Even under the most favorable circumstances it is rather a doubtful mode, as it will be found difficult to graduate the compression, and if large and irregular in shape, still more difficult to have the compression uniform over the entire mass. Electrolysis as a means of consolidating the vessels and thus obliterating the tumor, is still practiced by surgeons when practicable. If, however, we have no experience, and are not familiar with the strength of the electric current we are using, we are more likely to produce with our needles the effect of the actual cautery than the more desirable electrolytic action. I have, in two small nævi, used electrolysis with happy results; and even where I had the effect of the actual cautery, with rather profuse suppuration, I had no reason to complain of the results.

Feeling, however, that I can better impress members of the profession with my views as to the treatment of this disease and the mode of conducting it by the report of cases, I have selected half a dozen or more, not for any interest in any one particular case, but to illustrate some of the advantages of the different modes of treatment, and their adaptability to different stages or conditions of the disease.

CASE I.—Mr. H., of this city, consulted me ten months ago in reference to his child, six months old, for, as he called it, "a mother's mark." Upon examination I found a scarlet-colored spot on the left side of the nose, about the size of the head of a large pin. Neither father nor mother could tell me whether the mark had increased in size or not, but both were impressed with the fact that the redness was more intense, and the mark more prominent on the skin, when the child cried. I asked them to return with the child in a week, which they did, and I found that the nævus had doubled in size in one week. The next day I destroyed it by puncturing it at two points with a small needle at white heat. A small pledget of lint, wet in cold water, was applied to the burn, and secured by a piece of isinglass plaster, with instructions to reapply it if it came off. In three or four days the child returned—the plaster was removed, and there was not a vestige left of the little vascular growth. A few months later I saw the child, and nothing was left but a little cicatrix that very much resembled a small scar from chickenpox.

CASE II.—In 1879, a mulatto child, two months old, was brought to my office with a nævus just over the centre of the eyebrow. It was three or four times the size of the mark in Case I.; in every other particular it very much resembled it. I performed the very same operation as in Case I., making four instead of two punctures. Four or five days later I saw the child, and it was evident that the diseased vessels were only partially destroyed. My mistake was that I had not penetrated deep enough. The subcutaneous vessels were already involved. A second operation with a stouter needle, and two punctures extending through the thickness of the true skin to the subcutaneous vessels, was completely successful. I report this case to show the importance of always introducing the needles sufficiently deep to reach all the diseased vessels.

A successful vaccination would have cured Case I., but would not have cured Case II. Small silk or cotton thread, saturated with tincture of iron and passed through the nævus to the depth of the disease, would have been successful, but the suffering would have been much more intense and continued longer, and the result most likely would have been much more unsightly. Nitric acid and such like substances would have been successful, but the increased and long continued suffering, and the unsightly cicatrix which sometimes results from these caustics, have deterred me from their use. The next two cases will illustrate a form that I have often met with.

CASE III.—Mr. B., of Brooks county, consulted me in 1879 in reference to his son, four months old. Upon examination I found a tumor of purple cast, about the size of a large bean. The father stated that it had perceptibly increased in size for the past month or two and that it was much larger when the child cried than when it was quiet. The feel was cavernous and puffy, disappearing under pressure, and rapidly refilling and assuming its former size when the pressure was removed. The treatment, which was commenced at once, was the actual cautery with stout needles at white heat. I passed them into the mass a number of times, continuing it until

I felt confident that I had touched with the hot needles the entire diseased mass of vessels. Cold water dressing was at once applied and frequently renewed. In three or four days a free suppuration set in, and for two days was rather profuse. I never saw my little patient from the day he left my office—the seventh day after the operation—for three years and a half, and was surprised and gratified to find that the cicatrix, "the result of the operation," was hardly perceptible.

CASE IV.—Mary C., a mulatto woman, presented her child, one year and ten months old, at the College clinic, session 1875-76, for treatment. This case was similar to Case III., in the same locality and about one-fourth larger, with the same discoloration, so far as to the tumor proper, but in addition there was a scarlot spot the size of a pea just above the border of the lip. The mother stated that the red spot had existed since the birth of the child, but at birth was not so large—that the tumor or growth had been increasing in size for several months. I treated this case by the injection of the perchloride of iron, by means of a hypodermic syringe, depositing two or three drops in half a dozen different localities in the mass of vessels. There was slight suppuration at the point where I introduced the needle, which was through the red spot. For a few weeks I thought the operation was a success, but at the expiration of a month or five weeks, it was evident that the trouble was not relieved. Two more operations, similar to the first, with four weeks intervening, were necessary to effect a permanent cure. As will be seen, we adopted different plans of treatment in the two last cases reported. and that, although the cases were similar. the difference in the results was marked. The first was cured by one operation, and dismissed in a week; the other requiring three operations, and three months to effect the cure. There are but few mothers, however, who, if the two plans of treatment were suggested to them, but would select the injection of the perchloride of iron, with the idea that the suffering, both to themselves and children, would be much less, than with the actual cautery. But, from the report of the two cases, it is evident that the sufferings in the case treated by the actual cautery was much less than by the injection of the styptic. My experience is that nine times out of ten we will have the above results.

CASE V.—The son of Mr. S., of Fayette Co., eight years old, was brought to the city in 1869, to consult me in regard to a rather large, irregular and mixed nævus of the scalp, about the centre of the left parietal bone. He had had several hemorrhages, two of which were profuse. The growth presented two or three ulcerated surfaces, one of which was the size of a quarter of a dollar. The tumor was irregular in shape, the size of a turkey's egg or larger, with irregular projections. Two-thirds of the skin covering the tumor was involved in the disease. I decided to remove it with the ligature, and as the tumor was oblong and irregular and the greater portion of the skin involved, I used the subcutaneous ligature and made six segments. To do this, I armed a large curved needle with a double cord, one black (made so by immersing it in ink), and the other white; after elevating the tumor, the needle was passed through the sound skin on one side, passing entirely beneath the tumor and out through the sound skin on the other side—passing the needle through at right angles to the long axis of the tumor; the needle with cords was passed through in this way three times, thus dividing the growth into six segments. On one side all the loops of the white cord were cut, and on the other side all the loops of the black cord were cut; each pair of white ends were now tied securely on one side, and each pair of black ends on the other side were securely tied in the same way; and thus the six segments were effectually strangulated. In a few days the strangulated mass sloughed without the loss of blood or other unpleasant symptoms, and the patient had a rapid and permanent recovery.

The two following cases we have had illustrated by wood cuts taken from photographs, before and after the operation, to show the enormous size and the hideous deformity which we sometimes have from this apparently trivial commencement—a little discolored spot—and to show the possible beautiful results that may be obtained, even after such enormous growth, by one or more operations.

CASE VI.—June, 1859, Mr. H., of Gilmore county, consulted me in reference to his little daughter, two years old. He gave me the following history: The child at birth had a mother's mark just over the eyebrow, about the size of a pea. For a time it remained stationary, but three or four months after birth he noticed that it was growing—or swelling, as he expressed it; the growth was very slow up to three months ago, since which time it has been very rapid. Upon examination I found a tumor of large size, extending from an inch above the orbital process of the occipital bone, to a point on a line with the ala of the nose on the left side. From a superficial examination it presented the appearance as if the entire mass passed out of the orbital cavity. A more careful examination, however, made it evident that the eye and its appendages were intact. The eye could partially be seen by forcibly elevating the bag-like tumor, suspended over the eye. I could force my finger up sufficiently to feel the ball and to determine that it was not involved, but was fully impressed at the time, that the entire upper lid was involved. There were two discolored spots, about the size of a five-cent piece, just above the orbital process of frontal bone. After mature deliberation, I decided to attempt to destroy the enormous subcutaneous net-work of vessels, by a succession of operations with the actual cautery needles. One great object was to preserve the skin to the extent possible, and to attack the tumor by passing the needles through the discolored spots.

The little patient was made insensible with chloroform, and, with needles at white heat, I introduced twelve or fifteen through the discolored spots into different parts of the tumor. By the constant application of cold water, by means of a pledget of lint, and an occasional anodyne, the child suffered but very little, sleeping the greater portion of the time for twenty-four hours. Four other operations, similar in character,were performed at intervals, of from six to twelve weeks. The result was all that I could have expected. The eye was perfect, and the only defect was the upper lid which was a little stiff and did not close perfectly, but without a careful examination could not be detected unless she attempted to close her eyelids, when it was very perceptible. The cicatrices at the points where the needles were so frequently introduced—the discolored points—disfigured her to some extent. I proposed their removal, but the parents objected to the operation.*

CASE VII.—B. G., a colored man, of Clayton, Ala., twenty years old, consulted me at the clinic of the Atlanta Medical College, in January, 1880, for an enormous nævus of the upper lip. The history obtained from him was, that he had a lump, as he expressed it, and a discolored spot on his lip since his earliest recollection, but just at what point it commenced he could not tell; said that his mother told him he had a mark on his lip at birth. The extent and appearance presented by this enormous vascular tumor is better given by the wood-cuts, Figs. 1 and 2, than I could possibly give by words. Fig. 1, as will be seen, is a front view, and shows that the tumor not only involves the entire upper lip, but extends up, under and

* As above stated, the photographs were sent to have wood-cuts to illustrate this case, but when they arrived they were so defective that I returned them with the hope of getting others, but, after waiting as long as the publication could be deferred, I am forced to go to press without them.

above the ala of the nose, on the right side, under the septum, separating it from the vomer, and extending from a half to three-fourths of an inch in either nostril. The profile view, Fig. 2, was taken with a support, a thin piece of wood held between the teeth, and thus holding up the tumor that the entire bulk might be presented. Both show, in addition to the extensions above mentioned, an extension upon both sides of the cheek, some distance beyond the angles of the mouth. As can be readily seen from Fig. 2, he

FIG. I.—NÆVUS—CASE VII.—FRONT VIEW.

(From a Photograph.)

had several ulcerated points on different parts of the tumor, and also numerous cicatrices, the result of injuries received, and ulcers that had healed. He also stated that he had had innumerable hemorrhages, two of which were profuse—to the extent that, as he expressed it, "he bled to death;" that in the last, which occurred two months before I saw him, he was unconscious for forty-eight hours. He presented himself for treatment, and was willing to submit to anything that promised a chance of relief. It was evident that he could not live a great while in the condition above described—perhaps the very next hemorrhage would prove fatal, and might take place any day. And, then, to any one with ordinary sensibility, the great suffering from the numerous ulcerated points, the difficulty, annoyance and pain in taking food—there being a coustant dribbling of a sero-sanguineous fluid—and above all, the hideous appearance presented, made life unendurable.

After deciding to operate, the next question to be solved was, what operation or operations would most likely save his life, and at the same time make him most presentable after his recovery. I

FIG. 2.—NÆVUS—CASE VII.—PROFILE VIEW.

(From a Photograph.)

decided to attempt enucleation of the mass; not that I expected to remove all the diseased vessels, but to the extent practicable. In looking at the front view, Fig. 1, it will be seen that there was a considerable portion of the skin covering the tumor—more on the left than the right, as shown in the wood cut - that was comparatively healthy, that although it was studded with diseased and discolored points, still I thought it could be made serviceable in arresting hemorrhage in an emergency, and possibly be of use later in the formation of a lip. In profile view, Fig. 2, it can be seen that the mucous membrane presented a similar condition. In preparing for the operation, I had everything in readiness to meet any emergency: a full set of actual cautery irons—from needles up, and so arranged that I could have them at any desired heat; any quantity of well prepared lint, saturated with the muriated tincture of iron; ligatures, sutures. etc. Upon a careful examination it was found that while the veins predominated in the tumor, there were numerous tortuous arteries losing themselves in the mass of vessels.

FIG. 3.—NÆVUS—CASE VII.—AFTER OPERATION.

(From a Photograph.)

The coronary arteries on either side were more than twice their normal size. Feeling that no operation could be performed without the greatest risk, unless the supply of blood from these arteries could be controlled, I procured a clamp, one for either side, by which the circulation in these arteries could, to a very great extent, be under the control of an assistant.

After every detail was arranged the patient was partially etherized, and placed in a semi-reclining posture; the clamps were applied, and placed under the control of an assistant, whose duty it was to keep them in position and sufficiently tight to control the circulation of the superior coronary arteries. With a knife and a strong pair of curved scissors, I now rapidly enucleated the tumor in mass. When I say "enucleated the tumor," it is not strictly true, as much of the diseased mass was left, but the great bulk was removed, leaving two flaps—one made by cutting the mass from the skin, and the other from the mucous membrane. The projections of the tumor under the alæ, the vomer and vicinity, as well as the lateral projections in the cheeks, were left; and when the section was made, notwithstanding the fact that the coronary arteries were compressed, the hemorrhage was profuse. The flaps were separated by an assistant, and the actual cautery was applied to the portion of the tumor left, with the hope of arresting the profuse hemorrhage. Frequent applications of the actual cautery were made, and although the arteries were still compressed by the clamps the hemorrhage continued. Having failed with the red-hot iron to stay the flow of blood, I filled and packed the space between the two flaps of skin and mucous membrane, with lint saturated with the muriated tincture of iron, and rapidly and securely brought the edges of the flaps together with the continuous suture. After inspecting carefully the stump, as I called it, which was now with the lint more than one third the size of the original tumor, adding an occasional stitch to make it more secure, I removed the clamps, and I am sure the blood lost after packing it did not amount to as much as half an ounce.

While the loss of blood during the operation was considerable, still it was not so much as I feared would be lost. He came out of the operation with pretty fair pulse and in every particular much better than expected. Four days after the operation I removed the lint without hemorrhage, cleansed the wound with carbolic acid, leaving the flaps alone. Twelve days after the first operation I again etherized the patient, and with a number of stout strong needles the size of a shoemaker's awl, I thoroughly destroyed all that portion of the vascular mass left from the first operation. The needles were passed under the alæ of the nose, the vomer, and in fact every point where I could detect the disease. The needles were in every instance introduced between the flaps, and even where the flaps were punctured it was from the incised side. There was rather more hemorrhage in this operation than is usual with the needles; to the extent did it bleed, that I packed it as before, not however closing the flaps, as in first operation, but simply closing them with two or three interrupted sutures to hold the lint in position. The flaps and surrounding diseased tissue had, in seven weeks after the first operation, greatly contracted; to the extent had the disease been relieved that a few days later, seven weeks and a half after the first operation, the third and last operation was performed. The shape and appearance of the upper lip at this time was remarkable. It presented the appearance of anything but a lip. With a sharp knife the skin was detached from the malar bone, from the ala of the left side and vomer, and the incision so made that when brought together we had the result represented by Fig. 3. As will be seen in this wood cut, it was necessary to extend the incision into the nasal cavity, excising a portion of skin and mucous membrane. The patient was sufficiently recovered to leave for his home ten days after the last operation.

Case VIII.—In July, 1880, I was consulted by Mr. B , of Troup county, in reference to his daughter, six or seven years of age. At birth her mother informed me that she had the discolored marks the size of a five cent piece, on the right labium majus For several years she detected no change, but for the past two or three years they had been growing more or less rapidly. I found upon examination, the discolored spots as large as a quarter of a dollar, with a very extensive vascular tumor, involving the entire labium majus and the upper portion of the labium minus extending out towards the groin down into the perineum to near the anus, and extending out to the border of the muscles. As the father lived some distance from the city, and being a man of very limited means, he insisted that if possible, he very much desired that the cure should be effected at one operation. The little girl was etherized, and with a sufficient number of needles, about the size of a shoemaker's sewing awl, so arranged that one or two could be kept at white heat, the operation was commenced. Every portion of the tumor was attacked; the number of times the needles were introduced into the vascular mass, it is impossible to say, but several who witnessed the operation say that there were between sixty and eighty punctures. To save the skin to the extent possible, I sometimes introduced from ten to fifteen needles through the same puncture in the skin, passing them in different directions after entering the tumor. I have never seen the patient since the operation, but learn that she had a rather extensive suppuration, lasting for three weeks or more, and that she is entirely relieved of the vascular growth.

I could give many other cases of like character, but the above are sufficient to illustrate the principle that I so much desire to impress

—that is, that just as soon as it has been determined that the nævus, however small, is increasing in size, we should immediately adopt active measures to destroy it. To temporize and delay action will be doing our patients great injustice, as is fully demonstrated by the above reported cases.

## Figures and Tables

**Fig. 1. f1:**
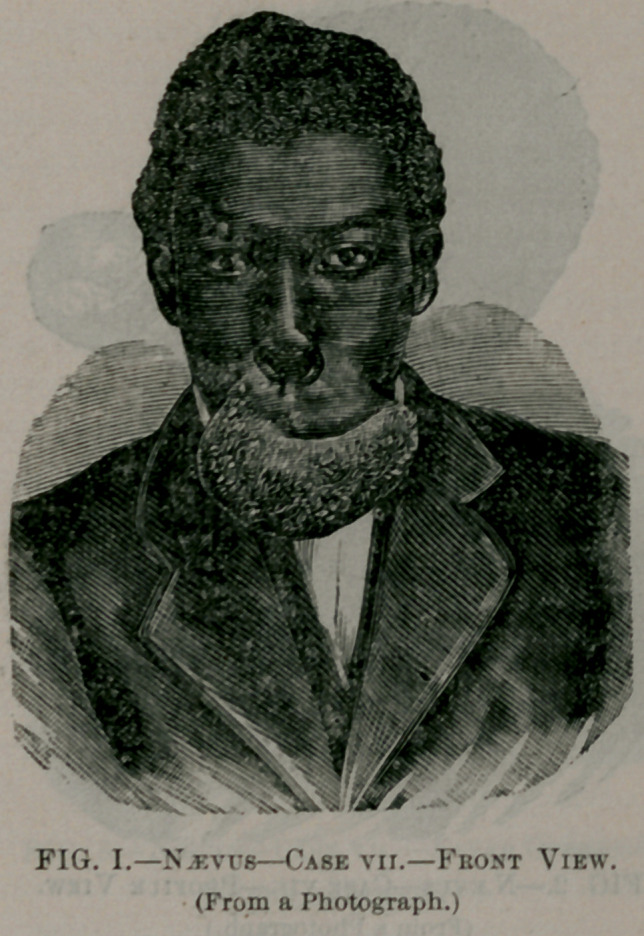


**Fig. 2. f2:**
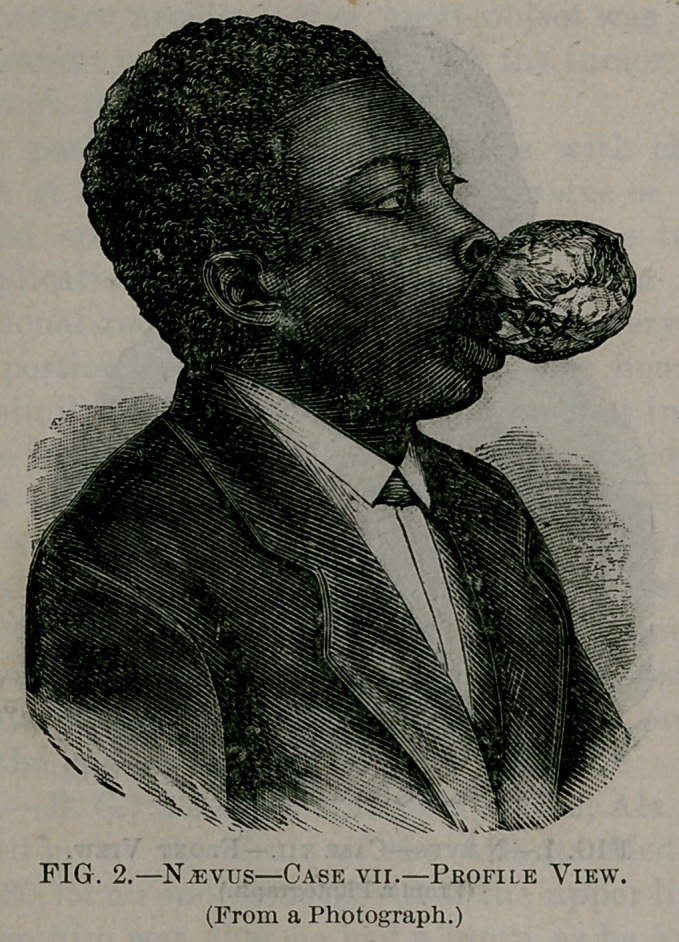


**Fig. 3. f3:**